# Phage therapy against *Pseudomonas aeruginosa* infections in a cystic fibrosis zebrafish model

**DOI:** 10.1038/s41598-018-37636-x

**Published:** 2019-02-06

**Authors:** Marco Cafora, Gianluca Deflorian, Francesca Forti, Laura Ferrari, Giorgio Binelli, Federica Briani, Daniela Ghisotti, Anna Pistocchi

**Affiliations:** 1 Dipartimento di Biotecnologie Mediche e Medicina Traslazionale, Università degli Studi di Milano – LITA, via Fratelli Cervi 93, 20090 Segrate, MI Italy; 2 Istituto FIRC di Oncologia Molecolare – IFOM, Via Adamello 16, 20139 Milano, Italy; 3 Dipartimento di Bioscienze, Università degli Studi di Milano, Via Celoria 26, 20133 Milano, Italy; 4 Dipartimento di Biotecnologie e Scienze della Vita, Università degli Studi dell’Insubria, Via J.H. Dunant 3, Varese, Italy

## Abstract

Cystic fibrosis (CF) is a hereditary disease due to mutations in the *CFTR* gene and causes mortality in humans mainly due to respiratory infections caused by *Pseudomonas aeruginosa*. In a previous work we used phage therapy, which is a treatment with a mix of phages, to actively counteract acute *P*. *aeruginosa* infections in mice and *Galleria mellonella* larvae. In this work we apply phage therapy to the treatment of *P*. *aeruginosa* PAO1 infections in a CF zebrafish model. The structure of the CFTR channel is evolutionary conserved between fish and mammals and *cftr*-loss-of-function zebrafish embryos show a phenotype that recapitulates the human disease, in particular with destruction of the pancreas. We show that phage therapy is able to decrease lethality, bacterial burden, and the pro-inflammatory response caused by PAO1 infection. In addition, phage administration relieves the constitutive inflammatory state of CF embryos. To our knowledge, this is the first time that phage therapy is used to cure *P*. *aeruginosa* infections in a CF animal model. We also find that the curative effect against PAO1 infections is improved by combining phages and antibiotic treatments, opening a useful therapeutic approach that could reduce antibiotic doses and time of administration.

## Introduction

One of the most serious health emergencies in the last decades is the reappearance of bacterial infections^1,2^. This serious fallout is a consequence of the rapid spread of resistance towards currently in use antibiotics among pathogenic bacteria together with the difficulty in discovering new effective antibiotics. In addition, the appearance and diffusion of multidrug resistant (MDR) isolates make the situation even worse^3^. Thus, alternative therapies are urgently needed and bacteriophages (phages), the natural enemies of bacteria, can be a possible solution. Compared to antibiotics, phages have several advantages: first, they infect only very specific bacterial hosts avoiding damage to healthy commensal microbiota^4^; second, phages are self-controlling their dose: they multiply when and where the target bacterial host strains are present, increasing their number at the infection site only as long as the target bacteria are eliminated^5^; lastly, phages are able to kill also MDR bacteria^6^.

The idea of using phages against bacteria is not new: the first attempts were made almost a century ago^7^. However, due to the lack of knowledge of the phage reproductive cycle, the therapy alternated successes and failures and, with the advent of antibiotics, phages were abandoned in the Western world unless for compassionate treatments^8,9^, although they are currently in use in Eastern world. Nowadays, many details of the reproduction of phages have been thoroughly clarified, which facilitate their use in therapy and guidelines have been suggested for preparation and use of phages as therapeutic agents^10^.

In the last years, an increasing number of reports on the effectiveness of phage therapy in controlling bacterial infections have been provided, ranging from *Klebsiella pneumoniae* pulmonary infections^11^, *Pseudomonas aeruginosa* keratitis^12^, or *P. aeruginosa* infected mice^4,13,14^. In a recent report^15^, we isolated and characterized virulent phages capable of infecting *P. aeruginosa*, and sequenced their genomes to exclude the presence of any gene potentially harmful in therapy. Different phages were mixed in a cocktail and used to treat *P. aeruginosa* acute infections in mouse and *Galleria mellonella* larvae. Phage therapy was successful in both model systems. Moreover, we found that the efficacy of the therapy was improved using a phage cocktail compared to the use of a single phage^15,16^, a likely consequence of the enlargement of the host range and of the reduced frequency of bacteria resistant to phages, as reported by Chadha *et al*.^17^.

*P*. *aeruginosa* infections are particularly serious in patients affected by cystic fibrosis (CF) being one of the major causes of mortality and morbidity^18^. Cystic fibrosis is a recessive genetic disease caused by mutation of the gene which encodes the cystic fibrosis transmembrane regulator (CFTR), a chloride ion channel^19^. Due to widespread CFTR protein channel distribution, CF affects multiple organs including the lung, gastrointestinal tract, liver, male reproductive tract and pancreas. One of the major complications in CF patients is chronic infection of the airways, principally caused by *P*. *aeruginosa*, and as a consequence, CF patients are subject to frequent antibiotic treatments to control the infections. The positive outcome of acute *P*. *aeruginosa* infections obtained by phage therapy encouraged us to further investigate its use in a CF background, and we chose zebrafish (*Danio rerio*) as a model system to validate it. Indeed, zebrafish represents a good model for CF as the zebrafish Cftr channel has a similar structure to the human CFTR^20^ and embryos with *cftr* knock-down present a specific sensitivity to infection with PAO1, in line with the susceptibility of CF patients to this bacterium^21,22^. Indeed, although fish do not have lung, the mainly affected organ by *P. aeruginosa* infection in CF patients, they have mucins, the proteins overexpressed in the lungs of CF patients. Zebrafish mucins are highly homologues to human mucins in terms of genomic and protein domain organization^23^. This observation, together with evidences of a development of *P*. *aeruginosa* microcolonies, the precursors of biofilm, in zebrafish^24^, make zebrafish a good model to study *P*. *aeruginosa* infection in all organs but lungs. Moreover, deregulation of *cftr* function in zebrafish causes a phenotype that mirrors other defects present in the human disease such as severe pancreatic dysfunction^25,26^, not observed in CF mouse model^27^ and hematopoietic defects that might explain the anaemia presented by CF patients^28^. Zebrafish possesses an additional advantage as it lacks an adaptive immune response for the first 4–6 weeks of life representing an ideal model for studying innate immunity^29^, which is the critical defence mechanism in human lung infections^30^. Indeed, it has been demonstrated that pathogen recognition and inflammation response through the release of cytokines occurs in similar manners in zebrafish and humans^31^.

In this work, using *cftr*-loss-of-function zebrafish embryos (CF embryos), we demonstrate that phage therapy is effective against *P*. *aeruginosa* infections. Moreover, we show that by combining phages and antibiotic treatments, the curative effect is improved suggesting that the administration of phages together with antibiotics could reduce antibiotic doses and time of administration.

## Results

### *Pseudomonas aeruginosa* PAO1 infection of zebrafish embryos

**Figure 1:**
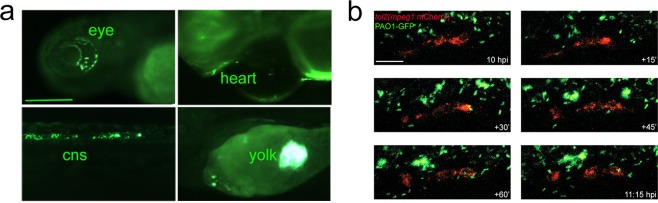
Infection of zebrafish embryos with PAO1. (**a**) PAO1-GFP injected bacteria were visualized in different districts of the embryo at 20 hpi. Cns: central nervous system. Scale bar indicates 100 μm. (**b**) Injection of the tol2(*mpeg1*:mcherry) plasmid allowed the visualization of red macrophages migrating towards green PAO-GFP bacteria. Starting from 10 hpi, different images of the same region of the trunk were taken every 15 minutes. Scale bar indicates 20 μm.

PAO1 infection was performed in zebrafish embryos at 48 hours post infection (hpi) by microinjecting into the duct of Cuvier approximately 30 colony forming units (cfu)/embryo, as previously described^32^. Bacterial dispersion within the embryo occurred immediately, as evaluated by disappearance of the dye tracer co-injected with the bacterial suspension. The distribution of fluorescent bacteria within the embryos was followed by microscopy: as reported in Fig. 1a, at 20 hpi bacteria could be visualized in different organs of the embryos, indicating that bacterial infection can spread in the whole embryo. Moreover, we verified that PAO1 infection was recognized by host immune system. By microinjecting the tol2(*mpeg1*:mCherry) construct that labels the macrophages, we observed their migration towards GFP positive PAO1 and that bacteria were engulfed by macrophages (Fig. 1b).

### Generation of a zebrafish CF model

**Figure 2:**
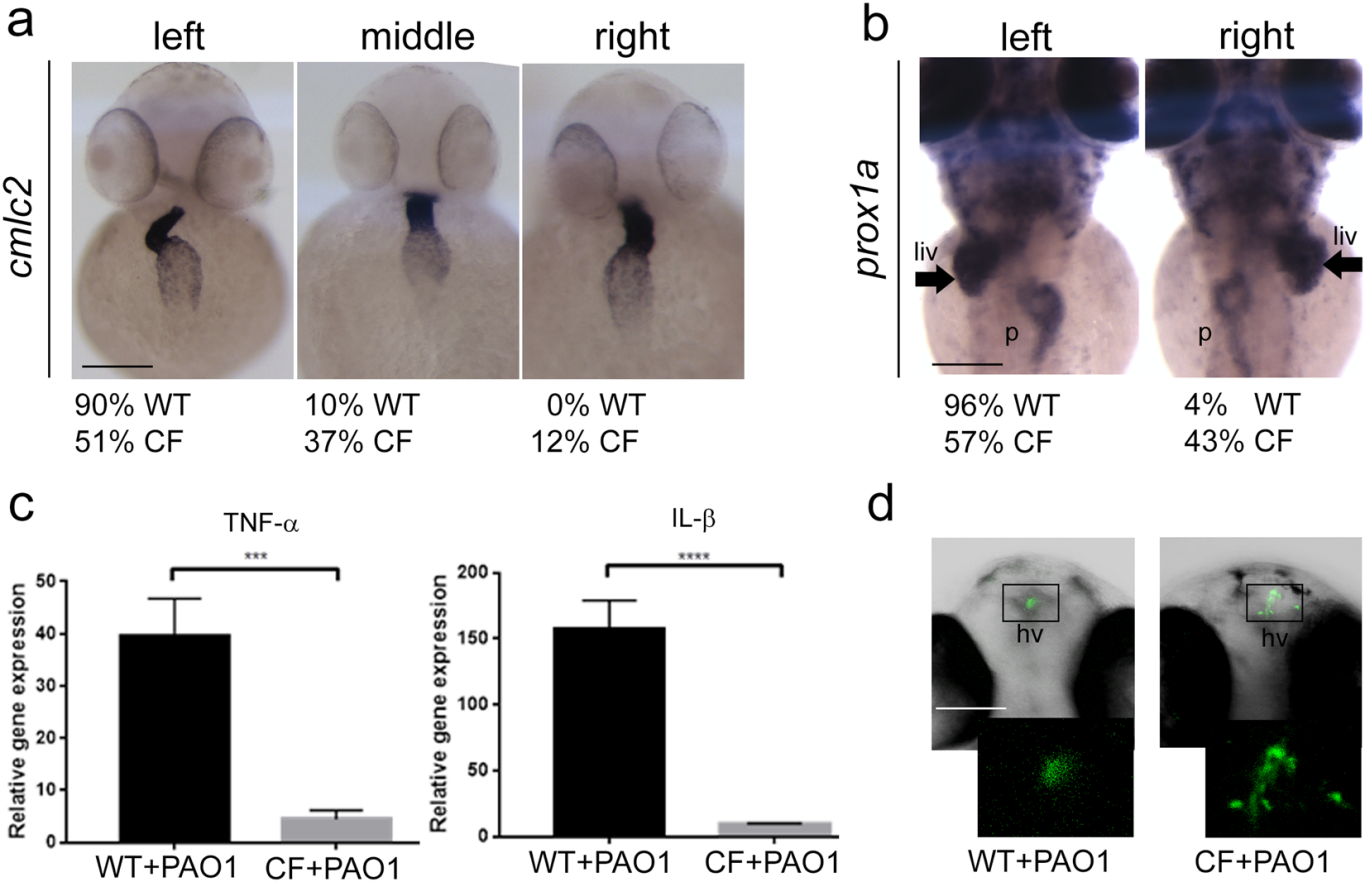
Validation of CF embryos. (**a**,**b**) Impaired internal organ position in CF injected embryos. (**a**) The normal left-looping of the heart (ventral view of the embryo, visualized by means of *cmlc2 in-situ* hybridization techniques), and (**b**) the normal left-position of the liver (arrows, dorsal view of the embryo visualized by means of *prox1a in-situ* hybridization techniques), were impaired in CF injected embryos in comparison to controls. Scale bars indicate 100 μm. liv: liver; p: pancreas. (**c**) Induction of TNF-α and IL-β. Expression levels, measured by RT-qPCR analyses at 8 hpi, in 48 hpf WT and CF embryos injected with PAO1 (+PAO1) are given relative to the average levels measured in mock injected controls. One tailed *t*-test: *t*_[4]_ = 7.62, *p* = 7.9E10^−4^ (TNF-α); *t*_[4]_ = 15.26, *p* = 5.4E10^−5^ (IL-β). (**d**) PAO1 microcolony formation in the hindbrain ventricle of WT and CF zebrafish embryos. The amount of microcolonies in the CF background at 18 hpi was higher compared to the WT siblings injected with the same amount of PAO1 (100 cfu). Scale bar indicates 100 μm. hv: hindbrain ventricle.

To obtain a CF background in which validating the efficacy of phage therapy against PAO1 infections, we generated *cftr*-loss-of-function zebrafish embryos (CF embryos), by injecting the *cftr* morpholinos previously used and characterized^21^. The embryos exhibited the phenotypes already described^21,25^. Indeed, by *in situ* hybridization analyses, we demonstrated that the position of the internal organs such as heart, liver and pancreas was impaired in CF embryos at 48 hours post fertilization (hpf). The heart, visualised by *cardiac myosin light chain* 2 (*cmlc2*) probe, normally is left-looping. Indeed, the percentages of WT embryos were: 90% left-looping, 10% middle and 0% right-looping heart (N = 50). At the contrary, in CF embryos the percentages were: 51% left-looping, 37% middle and 12% right-looping heart, indicating a defect in heart looping (Fig. 2a). Also the normally left-position of the liver, visualised by *prox1a* probe, was impaired in the CF: 96% left-liver in WT compared to 57% left-liver in CF embryos at 48 hpf (N = 50) (Fig. 2b). Moreover, the CF embryos presented a delayed immune response following bacterial infection in comparison to the WT, as shown by significantly reduced TNF-α and IL-β activation at 8 hpi (Fig. 2c). To ascertain that PAO1 was able to form the biofilm, typical of CF lung colonization, we injected about 100 cfu of PAO1 in the hindbrain ventricle of 24 hpf zebrafish embryos, as reported by Rocker and colleagues^24^, and observed bacterial colonization by time laps analyses at confocal microscopy (Suppl. Video S1). At 18 hpi several microcolonies were visualized in the embryos, indicating that PAO1 has started to form the biofilm. Interestingly, the area of microcolonies formed in CF embryos was higher than in WT embryos (Fig. 1d).

### Validation of the phage therapy against PAO1 infection in WT and CF zebrafish embryos

**Figure 3:**
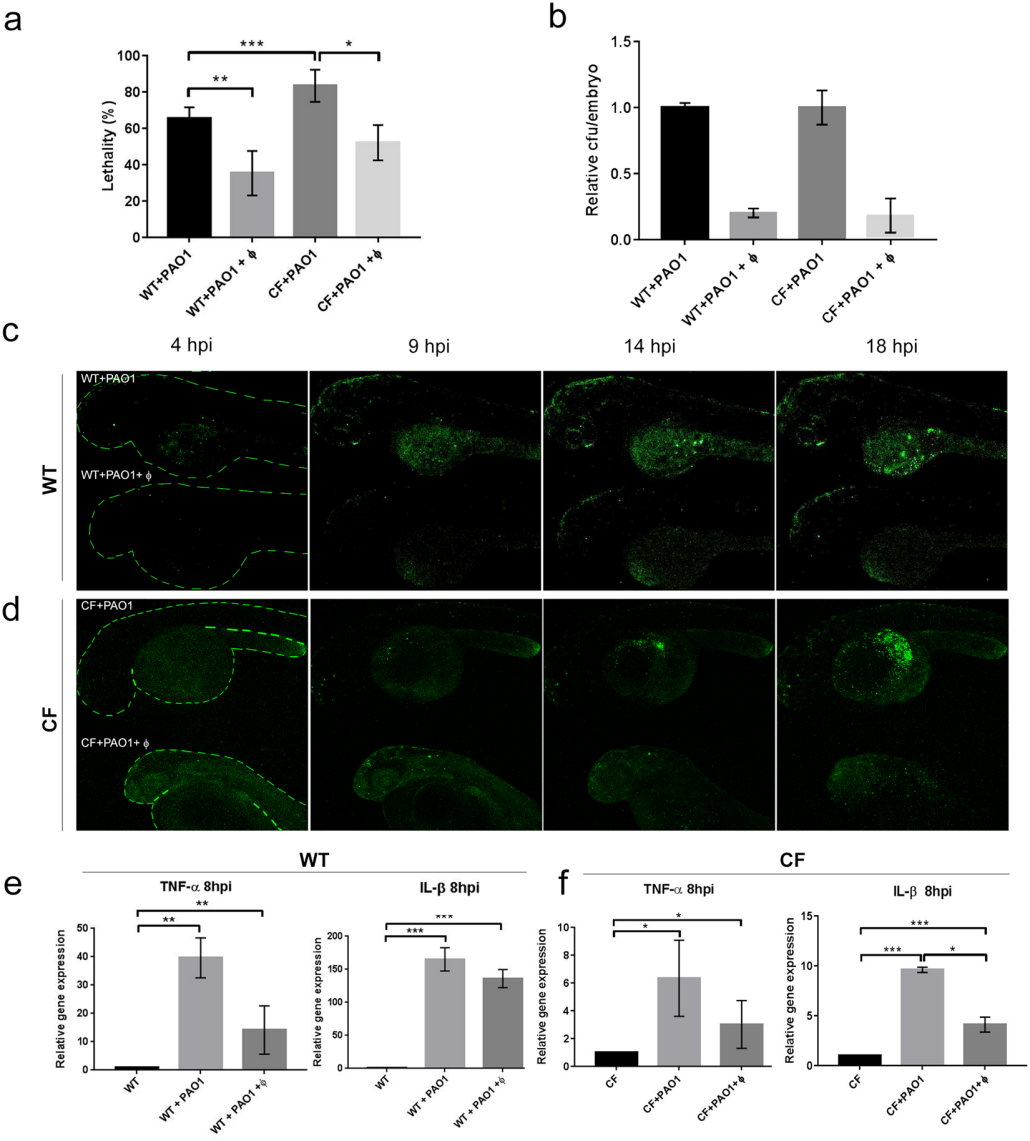
Efficacy of phage therapy in zebrafish. (**a**) Lethality at 20 hpi in WT and CF zebrafish embryos infected with PAO1 (+PAO1) and treated with the phage cocktail (+PAO1 + ϕ). The mean and SD reported are from six and four experiments, respectively, each with 25–40 embryos. Angular transformation was applied to percentage of lethality and one-way ANOVA followed by Duncan’s test was used to test for significance. (WT + PAO1) vs (WT + PAO1 + ϕ) *p* = 0.004; (WT + PAO1) vs (CF + PAO1) *p* = 0.0003; (CF + PAO1) vs (CF + PAO1 + ϕ) *p* = 0.018. (**b**) Bacterial burden in WT or CF embryos infected with PAO1 or PAO1 + ϕ. The relative percentage of cfu/embryo in PAO1 + ϕ vs PAO1 embryos are given. The mean and SD of three independent experiments is reported. (**c**,**d**) Progression of the infection in WT (**c**) and CF (**d**) embryos following PAO1 injection (upper embryo) and efficacy of the phage therapy in PAO1 + ϕ injected embryos (bottom embryo) at 4, 9, 14 and 18 hpi. (**e**) Expression levels of the TNF-α and IL-β genes measured by RT-qPCR at 8 hpi in WT (**e**) and CF (**f**) embryos injected at 48 hpf. The mean and SD of four experiments are reported. Statistical significance was assessed by ANOVA followed by Duncan’s test: (**e**) for TNF-α, (WT) vs (WT + PAO1) *p* = 0.0018; (WT) vs (WT + PAO1 + ϕ) *p* = 0.007; (WT + PAO1) vs (WT + PAO1 + ϕ) *p* = 0.22 n.s.; for IL-β, (WT) vs (WT + PAO1) *p* = 0.0002; (WT) vs (WT + PAO1 + ϕ) *p* = 0.0001; (WT + PAO1) vs (WT + PAO1 + ϕ) *p* = 0.94 n.s.; (**f**) for TNF-α, (CF) vs (CF + PAO1) *p* = 0.015; (CF) vs (CF + PAO1 + ϕ) *p* = 0.019; (CF + PAO1) vs (CF + PAO1 + ϕ) *p* = 0.77 n.s.; for IL-β, (CF) vs (CF + PAO1) *p* = 0.00014; (CF) vs (CF + PAO1 + ϕ) *p* = 0.0007; (CF + PAO1) vs (CF + PAO1 + ϕ) *p* = 0.031. For simplicity sake, in the figures *p < 0.05; **p < 0.01, ***p < 0.001.

PAO1 infection on WT and CF zebrafish embryos was performed as described above with injection in the duct of Cuvier at 48 hpf of approximately 30 cfu/embryo, a dose that caused 50% lethality after 20 hpi (Suppl. Fig. S1). For each treatment, 35–40 embryos were injected and the experiment was repeated at least fourfold. To compare the susceptibility to PAO1 infections of WT and CF, we infected both types of embryos and analysed the lethality at 20 hpi. As expected, the CF embryos were more susceptible to PAO1 infection as they exhibited a highly significant increased lethality in comparison to WT (83 ± 9% vs 66 ± 6%, respectively) at 20 hpi (Fig. 3a).

Four phages of our collection, able to infect PAO1 strain, were chosen to be mixed in a cocktail. Description of the phages is reported in Table S1. We preliminarily assess the activity of the phage cocktail in the treatment of PAO1 infection in *G*. *mellonella* larvae. We observed that lethality of PAO1 infected larvae at 20 hpi was significantly reduced from 66 ± 3% to 46 ± 6% when treated with the phage cocktail (Suppl. Fig. S2), thus confirming that the phage cocktail was active against PAO1 acute infections.

Then, to test the efficacy of phage therapy in zebrafish, we injected the same phage cocktail in PAO1-infected WT embryos; an inoculum of 2 nl of the phage cocktail, containing approximately 300–500 plaque forming units (pfu)/embryo, was injected in the yolk sac of PAO1-infected embryos (PAO1 + ϕ) at two different time points: about 30 min (early) and 7 hours (late) after bacterial injection. A highly significant reduction in terms of embryo lethality was observed with either injection time: lethality was reduced from 70 ± 12% to 42 ± 9% and to 38 ± 3% in early and late injection, respectively (Suppl. Fig. S3). Since no significant difference was observed, further analyses were conducted with early infection time.

Phage therapy against PAO1 infection was applied to both WT and CF embryos; a significant reduction of lethality was observed (from 66 ± 6% to 35 ± 12% for the WT and from 83 ± 9% to 52 ± 7% for CF embryos Fig. 3a), indicating that phage therapy is effective in zebrafish both in a WT and in a CF background. We also determined the bacterial burden in WT and CF embryos at 8 hpi following phage administration: a reduction of average cfu/embryo to about 20% were observed after phage treatment (Fig. 3b). To follow the progression of PAO1 infection and the efficacy of phage therapy, we performed time laps analyses of WT and CF embryos injected with PAO1 and with PAO1 and the phages. As shown in Fig. 3c, in WT + PAO1 injected embryo (upper), the fluorescence increased at 14 hpi and 18 hpi, indicating that the GFP-positive bacteria multiply, whereas in the WT + PAO1 + ϕ embryo (bottom) the fluorescence did not increase, likely as an effect of the phage anti-bacterial activity. Same results in terms of phage therapy efficacy were obtained in the CF background but, as expected, the levels of bacterial infection in CF + PAO1 embryos was higher in comparison to WT + PAO1 embryos (Fig. 3d, and Videos S2 and S3). We also checked the pro-inflammatory response measuring the expression of TNF-α and IL-β cytokines by RT-qPCR (Fig. 3e,f). The pro-inflammatory immune response was activated in both WT and CF infections (WT + PAO1 and CF + PAO1) at 8 hpi; injection of the phage cocktail caused a slight reduction of cytokine expression that was significant only for IL-β in a CF background.

### Analysis of phage effects on immune response

**Figure 4:**
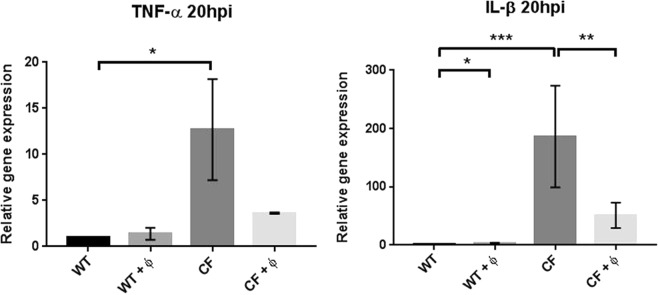
TNF-α and IL-β expression in phage injected WT and CF embryos. Expression levels of the TNF-α and IL-β genes measured by RT-qPCR at 20 hpi in WT, WT + ϕ, CF and CF + ϕ embryos injected at 48 hpf. The mean and SD of four experiments are reported. Statistical significance was assessed by ANOVA followed by Duncan’s test: for TNF-α, (WT) vs (WT + ϕ) *p* = 0.96 n.s.; (WT) vs (CF) *p* = 0.022; (CF) vs (CF + ϕ) *p* = 0.23 n.s. For IL-β, (WT) vs (WT + ϕ) *p* = 0.020; (WT) vs (CF) *p* = 0.00007; (CF) vs (CF + ϕ) *p* = 0.009.

In the absence of PAO1 bacterial infection, to better understand if phages might induce *per se* an immune reaction, we measured the expression of TNF-α and IL-β in WT and CF embryos without (WT and CF) or after phage administration (WT + ϕ and CF + ϕ) at 20 hpi. Interestingly, we found that the expression of TNF-α and IL-β was significantly higher in CF embryos with respect to WT (Fig. 4). These data indicate that the reduced activity of Cftr, *per se*, determines a constitutive inflammatory state^33–35^. To our surprise, in CF + ϕ embryos, we observed reduced expression of both cytokines, suggesting that the phage cocktail may have a beneficial effect against the constitutive inflammation of CF embryos (Fig. 4). It is to note that an increased expression of IL-β, but not of TNF-α, was observed also in WT + ϕ embryos.

### Combined effect of phages and antibiotics therapy

**Figure 5:**
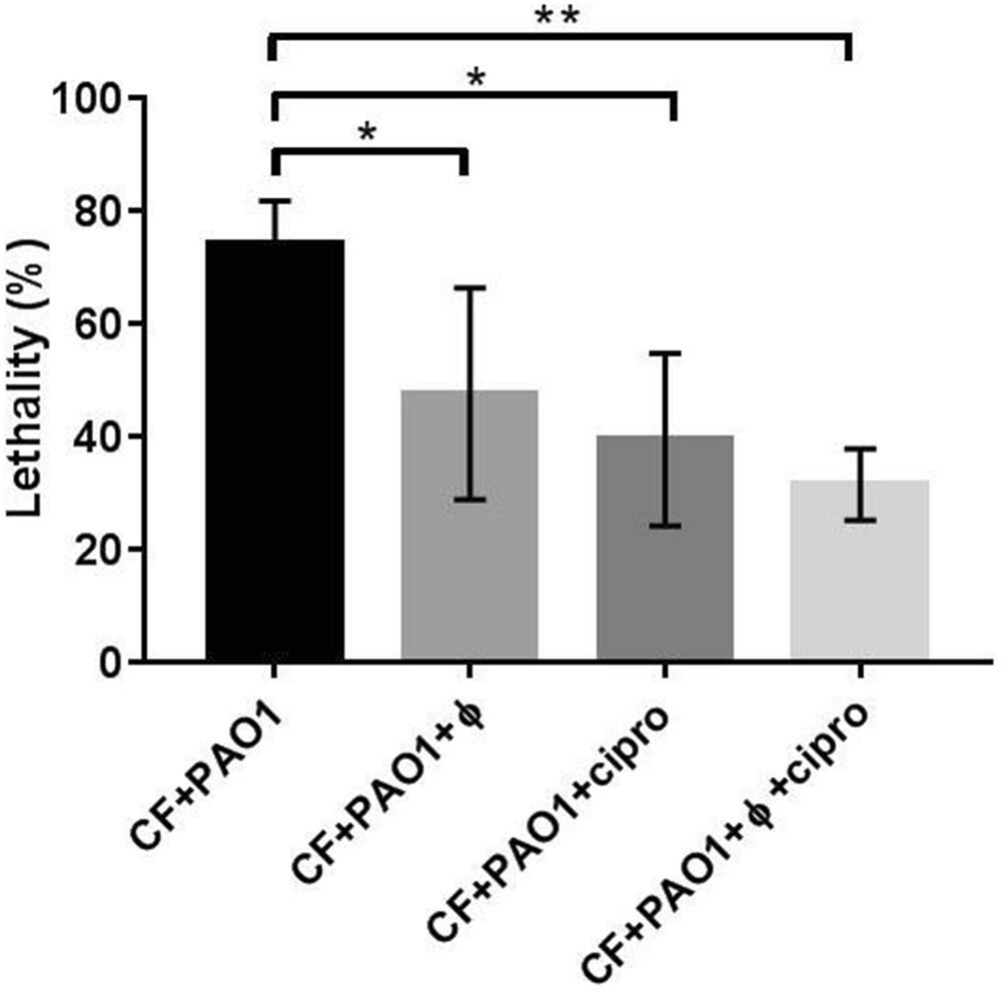
Combination of phage therapy and ciprofloxacin treatment in CF embryos. 48 hpf embryos were injected with PAO1 (CF + PAO1), followed by injection of the phage cocktail (CF + PAO + ϕ) or incubation in fish water containing 100 μg/ml ciprofloxacin (CF + PAO + cipro) or both (CF + PAO + ϕ + cipro). Lethality was determined at 20 hpi. The mean and SD of three experiments is reported, each with 25–40 embryos/treatment. Angular transformation was applied to percentage of lethality and one-way ANOVA followed by Dunnett’s test was used. *p*-values are reported for each treatment compared against CF + PAO1: CF + PAO + ϕ, *p* = 0.043; CF + PAO + Cipro, *p* = 0.014; CF + PAO + ϕ + Cipro, *p* = 0.005.

Since one of the main problems in CF patients is chronic bacterial infection and the development of antibiotic resistance, we tested the effects of combining phage therapy and antibiotic treatment by incubating CF + PAO1 embryos in fish water containing 100 μg/ml of ciprofloxacin (cipro), an antibiotic commonly used against *P*. *aeruginosa* infections. CF embryos infected with PAO1 and treated either with the antibiotic (PAO1 + cipro) or the phage cocktail (PAO1 + ϕ) showed reduced lethality in comparison to CF + PAO1 embryos. Interestingly, the combined treatment with phages and ciprofloxacin (CF + PAO1 + ϕ + cipro) further reduced the lethality in comparison to embryos treated with either one of the two (Fig. 5).

## Discussion

In this work, we demonstrated that phage therapy is able to fight *P*. *aeruginosa* infections in zebrafish embryos. Zebrafish embryos are gaining favour as infection models for pathogens like *P*. *aeruginosa*^36,37^. Recently, phage therapy has been applied to cure experimentally induced zebrafish infections by the fish pathogen *Aeromonas hydrophila*^38^. We recently showed that phage therapy cures acute respiratory *P*. *aeruginosa* infection in mouse and delays lethality in the wax moth *G*. *mellonella* larvae^15^. With this work, we enlarge the repertoire of infection models in which phage therapy against *P*. *aeruginosa* has been tested and proved to be effective, a relevant issue considering the wide variability of virulence shown by *P*. *aeruginosa* strains in different hosts^39^.

The usage of GFP-expressing bacteria coupled with transparency of zebrafish embryos allowed us to follow the bacterial infection in real time. Indeed, we observed that in the infected embryos that survive at 20 hpi, GFP-positive bacterial foci were present in different embryo districts indicating that the bacteria injected into the duct of Cuvier spread in the whole embryo and infect different organs. Furthermore, bacteria multiply in the embryo as shown by time laps analyses of PAO1 injected embryos (see Fig. 3c,d and Videos S2 and S3) and initiate microcolonies formation, the precursors of biofilm.

In our experiments, a very low inoculum of bacteria (30 cfu/embryo) was lethal for zebrafish embryos. This result was different from previous observations done by Llamas and Van der Sar and colleagues^36^ that performed the same type of infection with a higher dose of bacteria (over 500 cfu/embryo). PAO1 bacterial strain used for the infection and the protocols were the same, therefore we hypothesise that the different response to the infection might be due to differences accumulated in the PAO1 strains, commonly used in different laboratories, as previously demostrated^40^.

We found that phage therapy is effective against *P*. *aeruginosa* infections also in a CF zebrafish model generated through the *cftr*-loss-of-function technique. To our knowledge, this is the first time that phage therapy is successfully applied to an *in vivo* CF model system. To investigate the pathophysiology of CF several animal models have been developed including mouse, pig, and ferret^41–43^. Mouse models develop intestinal obstruction similar to CF patients, but fail to develop spontaneous lung disease^44^ and present only mild disease in the pancreas, likely due to compensatory calcium activated chloride channels^45^. The ferret and pig models of CF develop the pathophysiology characteristic of CF in many organs including lung and pancreas^43^, but they presented limited accessibility of developmental stages, high animal husbandry costs, and the impracticability of genetic analyses. Although devoid of lungs that are the main organs affected by *P. aeruginosa* infections in CF patients, zebrafish recently emerged as a powerful genetic model system to better understand CF onset and to develop new pharmacological treatments^21,26^. Indeed, the genetically tractability of zebrafish embryos to precisely analyse gene function, their optically transparency which facilitates the visualization of development or infection progression in real time, make zebrafish a suitable CF model, and the lack of an adaptive immune response in embryos until 30 days^29^, makes zebrafish an ideal model for studying innate immunity, which is the critical defence mechanism in human lung infections^46,47^. As already demonstrated, the CF background is more sensitive to *P*. *aeruginosa* PAO1 infections than the WT^21^. Indeed, we observed higher lethality and increased bacterial proliferation in CF + PAO1 embryos in comparison to WT + PAO1. In addition, after bacterial infection, CF embryos exhibit a delay in pro-inflammatory response in comparison to WT, suggesting an impairment of immune-response due to the absence of Cftr function.

Interestingly, we also observed that in the absence of induced PAO1 infection, in CF zebrafish embryos both TNF-α and IL-β cytokines were significantly activated in comparison to WT, suggesting that the lack of Cftr function, *per se*, causes a constitutive inflammatory state similar to the one present in human CF patients^33–35^. Indeed, previous studies using epithelial cells from CF patients have shown excessive expression of pro-inflammatory cytokines in absence of CFTR function^47,48^. We also demonstrated that in a CF background, the administration of the phage cocktail without bacterial infection, reduced TNF-α and IL-β expression to a level more similar to WT embryos. It might be that phages, besides counteracting bacterial infections by killing bacteria, may display a secondary function, acting as immunomodulators. Indeed, the constitutive inflammatory state of CF embryos was reduced with phage injection, while in the WT sibling the phage injection determines an increase of cytokines expression. This suggests that phages, *per se*, might activate a mild immune response but, when a constitutive inflammation is present as in the CF background, the final effect of phages is not pro-inflammatory. This hypothesis was raised previously by Gorsky and colleagues that reported that phages bind to mammalian cells, including lymphocytes, and produce immunosuppressive effects that enhance allogeneic skin allograft survival in mice^49^. Moreover, those effects were paralleled by phage-mediated inhibition of alloantigen-induced human T and B-cell responses *in vitro*, as well as specific antibody production in mice^50^. Thus, the potential of using phages as anti-inflammatory agents in CF patients might be evaluated.

In CF zebrafish embryos we observed positive interaction between phage and antibiotic therapy, as lethality was reduced when phage and ciprofloxacin were administered in combination to PAO1 infected embryos with respect to individual administrations. In other studies the combined action of phages and antibiotics has been proven to be helpful to control infections by different pathogens, including multiresistant strains^51^. Thus, the combination of phage therapy and antibiotic administration appears as a promising therapeutic approach, especially in order to reduce antibiotic doses and treatment duration^52^.

## Methods

### Bacterial strain

*P*. *aeruginosa* PAO1-GFP (indicated as PAO1) strain was constructed in our lab by transformation of PAO1 with a plasmid conferring carbenicillin resistance and expressing the GFP^15^. The strain can be observed by fluorescent microscopy. For the infection, PAO1 cultures were grown at 37 °C with shaking to OD_600_ = 0.5, corresponding to about 5 × 10^8^ cfu/ml in LD broth^53^ added with carbenicillin (300 μg/ml), pelleted and resuspended in the same volume of physiological solution. Dilutions were used for microinjections in zebrafish embryos.

### Phage cocktail

Virulent phages that infect PAO1 used throughout this work were isolated and characterized previously^15^. Four phages were selected: two Podoviridae, GenBank accession numbers vB_PaeP_PYO2, MF490236, and vB_PaeP_DEV, MF490238, and two Myoviridae, vB_PaeM_E215, MF490241, and vB_PaeM_E217, MF490240. Details of phages are reported in Suppl. Table S1. Each phage lysate was grown and purified as described by Forti *et al*.^15^; briefly, high-titer lysates of PAO1 cultures were filtrated with 1.2 μm diameter filters, incubated with DNase (1 μg/ml) and RNase (1 μg/ml), PEG precipitated, purified twice by cesium chloride ultracentrifugation, and dialyzed against TN buffer (10 mM Tris, 150 mM NaCl, pH 7), before endotoxin removal (EndoTrap HD, Hyglos, Germany) followed by measure of the endotoxin level by the LAL Chromogenic Endotoxin Quantitation (Pierce). The phage cocktail was assembled by mixing four phage preparations at the same pfu/ml immediately before each experiment to ensure accurate phage titres.

### Zebrafish model

Zebrafish (*Danio rerio*) embryos were raised and maintained under standard conditions in the fish facility of Cogentech s.c.a.r.l. (Aut. Prot. n. 007894 - 29/05/2018), via Adamello 16 - 20139 Milan (Italy). According to international (EU Directive 2010/63/EU) and national guidelines (Italian decree 4th March 2014, n. 26) on the protection of animals used for scientific purposes. Experiments with zebrafish are subject to regulations for animal experiments from 120 hpf onwards^54^. In this work we used exclusively zebrafish embryos up to 120 hpf. Zebrafish AB strains obtained from the Wilson lab, University College London, London, United Kingdom were maintained at 28 °C on a 14 h light/10 h dark cycle. Embryos were collected by natural spawning, staged according to Kimmel *et al*.^55^ and raised at 28 °C in fish water (Instant Ocean, 0,1% Methylene Blue) in Petri dishes, according to established techniques. After 24 hpf, to prevent pigmentation 0,003% 1-phenyl-2-thiourea (PTU, Sigma-Aldrich, Saint Louis, Missouri, USA) was added to the fish water. Embryos were washed, dechorionated and anaesthetized with 0.016% tricaine (Ethyl 3-aminobenzoate methanesulfonate salt; Sigma-Aldrich), before observations and picture acquisitions. Antibiotic administration was performed by adding ciprofloxacin directly into the fish water at a concentration of 100 μg/ml immediately after the bacterial injection. For *in situ* hybridization analyses, embryos were fixed overnight in 4% paraformaldehyde (Sigma-Aldrich) in Phosphate Buffer Saline (PBS; Sigma-Aldrich) at 4 °C, then dehydrated stepwise to methanol and stored at −20 °C.

### *cftr*-loss-of-function zebrafish embryos (CF embryos)

Antisense oligonucleotide morpholino injections were carried out on 1- to 2-cell stage embryos; the dye tracer rhodamine dextran was also co-injected when necessary. To repress *cftr* mRNA translation, the *cftr*-ATG-MO and *cftr*-splice-MO (Gene Tools LLC, Philomath, OR) were used as previously described^21^. Morpholinos were injected in 1x Danieau buffer (pH 7, 6). As control (WT), we injected a standard control morpholino oligonucleotide that has no target in zebrafish (Gene Tools LLC).

### Infection of zebrafish embryos with PAO1 and phages

PAO1 infection was performed in zebrafish embryos at 48 hpf by microinjecting 2 nl of bacterial suspension containing approximately 30 cfu/embryo into the duct of Cuvier, as previously described^32^. Evaluation of bacterial infection was performed following the guidelines of Takaki and colleagues^56^. To titre the injected bacteria, the same volume of the bacterial suspension was plated to determine the cfu; the titre of the injected bacteria was given by the average of eight independent measures. The phage cocktail (2 nl at 5 × 10^8^ pfu/ml) was microinjected in the yolk after bacterial injection, and the phage titre calculated in the same way as done for bacteria. Different time of injection of the phage cocktail were tested: early (about 30 min) and late (7 h) with similar results. In most experiments, we used early injection for practical reasons. In each experiment 25–40 embryos were injected for any single treatment and each experiment was repeated at least three times.

Guidelines to visualize host-bacterial interaction were followed^57^: macrophages were visualized with the injection of the tol2(*mpeg1*:mCherry) construct at one-cell stage as described by Ellet and colleagues^58^.

To visualise the microcolony formation, about 100 cfu of PAO1 were injected in the hindbrain ventricle of 24 hpf zebrafish embryos as performed by Rocker and colleagues^24^. We considered microcolonies the bacterial aggregates with an area >5 μm^2^.

### Determination of bacterial burden

PAO1 injected embryos were incubated at 28 °C for 8 hpi. 20 embryos in 3 different experiments were mechanically homogenized by means of an insulin syringe and the resulting homogenate serially diluted and plated to calculate the bacterial titer. To limit the growth of other bacterial strains, present in the embryos, ampicillin (100 μg/ml) was added to the medium to select for the naturally amp-resistant PAO1 strain.

### Determination of the expression level of pro-inflammatory cytokines genes

The expression level of TNF-α and IL-β genes were determined by reverse transcription-PCR and real-time quantitative-PCR (RT-qPCR) assays. Total RNA was extracted from zebrafish embryos at 8 hpi and 20 hpi using Trizol reagent (Life Technologies, Carlsbad, CA, USA) according to the producer’s instructions. After treatment with DNase I RNase-free (Roche Diagnostics, Basel, Swiss) to avoid possible genomic contamination, 1 μg of RNA was reverse-transcribed using the “ImProm-II™ Reverse Transcription System” (Promega, Madison, Wisconsin USA) and a mixture of oligo(dT) and random primers according to manufacturer’s instructions. qPCRs were carried out in a total volume of 20 µl containing 1X iQ SYBR Green Super Mix (Promega), using proper amount of the RT reaction. qPCRs were performed using the BioRad iCycler iQ Real Time Detection System (BioRad, Hercules, CA, USA). Thermocycling conditions were: 95 °C for 10 min, 95 °C for 10 s, and 55 °C for 30 s. All reactions were performed in triplicate for 40 cycles.

For normalization purposes, *rpl8* expression levels were tested in parallel with the gene of interest. The following primers were used:

*TNF*-α Fw 5′-TGCTTCACGCTCCATAAGACC3′;

*TNF*-α Rev 5′-CAAGCCACCTGAAGAAAAGG-3′;

*IL1*-β Fw 5′-TGGACTTCGCAGCACAAAATG-3′;

*IL1*-β Rev 5′-CGTTCACTTCACGCTCTTGGATG-3′;

*rpl8* Fw 5′-CTCCGTCTTCAAAGCCCAT-3′;

*rpl8* Rev5′-TCCTTCACGATCCCCTTGAT-3′.

### *In situ* hybridization analyses

Whole mount *in situ* hybridization (WISH) experiments were carried out as described by Thisse and colleague^59^. Antisense riboprobes were previously *in vitro* labelled with digoxigenin (Roche Diagnostics). *cmlc2 (myl7*, Zebrafish Information Network) and *prox1* probes have been previously described^60^. Images of embryos and sections were acquired using a microscope equipped with a digital camera with LAS Leica imaging software (Leica, Wetzlar, Germany). Images were processed using the Adobe Photoshop software and when necessary, different focal images planes of the same image were took separately and later merged in a single image.

### Imaging

For live confocal imaging, embryos at 54 hpf were mounted in a glass-bottom Petri dish with 1,5% low-melting agarose in fish water with PTU and the anaesthetic Tricaine, and immediately imaged on an SP2 confocal inverted microscope (Leica, Wetzlar, Germany) with an HC PL APO 10× objective and 488 nm argon laser for GFP and 587 nm for mcherry. Series of 300 μm z-stacks every 15 minutes were acquired. The frames resulted from confocal microscope acquisition were assembled and converted to an.avi movie using ImageJ software. When necessary, images were cut and reassembled accordingly to the proper orientation and disposition of the embryos.

### Statistical analyses

Angular transformation was applied to lethality data and transformed data analyzed by Student’s *t* or ANOVA, followed by post-hoc Duncan’s or Dunnett’s test, when required. The expression level of cytokines genes was analyzed by Student’s *t* or ANOVA. All analyses were run on STATISTICA v. 7.0 software.

## Supplementary information

Supplementary Materials

Video S1

Video S2

Video S3

## Data Availability

The datasets generated during and/or analysed during the current study are available from the corresponding author on reasonable request.
