# Supplementary material for: Phage therapy against Pseudomonas aeruginosa infections in a cystic fibrosis zebrafish model
Source: Sci Rep. 2019 Feb 6;9:1527. doi: 10.1038/s41598-018-37636-x (PMC6365511; doi:10.1038/s41598-018-37636-x)
Supplement: Supplementary file 1 — Supplementary Materials [file 41598_2018_37636_MOESM1_ESM.pdf]

Phage therapy against *Pseudomonas aeruginosa* infections in a cystic fibrosis zebrafish model

Marco Cafora[a#], Gianluca Deflorian[b#], Francesca Forti[c], Laura Ferrari[b], Giorgio Binelli[d], Federica Briani[c], Daniela Ghisotti[c], Anna Pistocchi[a*]

[a]Dipartimento di Biotecnologie Mediche e Medicina Traslazionale – Università degli Studi di Milano – LITA – via Fratelli Cervi 93 – 20090 Segrate (MI) - Italy
[b]Istituto FIRC di Oncologia Molecolare – IFOM – Via Adamello 16 – 20139 Milano - Italy
[c]Dipartimento di Bioscienze - Università degli Studi di Milano - Via Celoria 26 - 20133 Milano – Italy
[d]Dipartimento di Biotecnologie e Scienze della Vita - Università degli Studi dell'Insubria - Via J.H. Dunant 3 – Varese- Italy.

[#]These authors equally contributed to this work
*Corresponding author Anna Pistocchi email: anna.pistocchi@unimi.it
Dipartimento di Biotecnologie Mediche e Medicina Traslazionale, Università degli Studi di Milano. LITA, Via Fratelli Cervi 93, 20090, Segrate (MI), Italy.

SUPPLEMENTARY MATERIALS

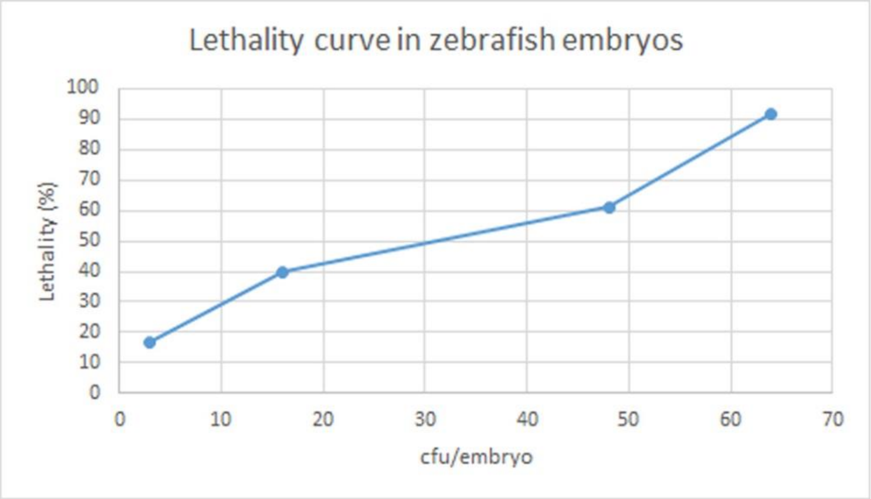

Fig. S1. Determination of LD_{50} in 48 hpf zebrafish embryos. 48 hpf zebrafish embryos were microinjected with 2 nl of a culture of PAO1 containing increasing number of bacteria (cfu/embryo). Lethality of the embryos was observed at 20 hpi.

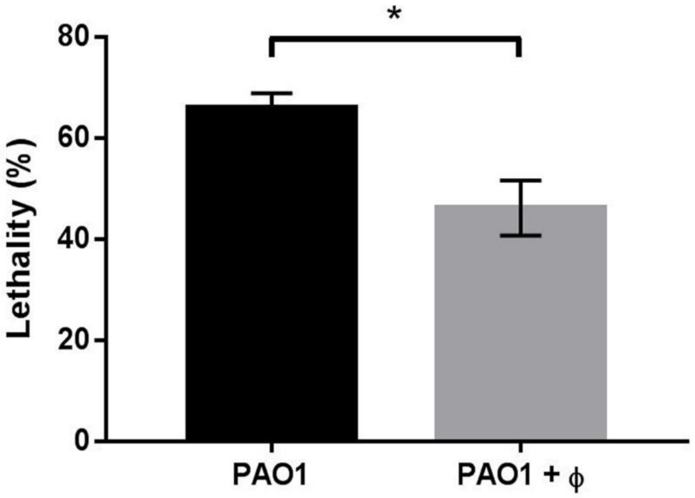

Fig. S2. Phage therapy in *G. mellonella* larvae. The experiment was performed as described in Forti et al. (2018). *Galleria mellonella* larvae were infected with 10 μl of *P. aeruginosa* PAO1 bacterial suspension, containing about 30 cfu, resuspended in physiological solution. The injection was delivered into the larvae haemolymph behind the last proleg. 1 h later larvae were inoculated behind the last proleg on the opposite side with 10 μl of physiological solution (PAO1) or 10 μl of the 4-phage cocktail at 1500 pfu (PAO1+ϕ). The phage cocktail was composed by equal amounts of four virulent phages (two Podoviridae and two Myoviridae), able to infect *in vitro P. aeruginosa* PAO1; the titre of the phages in 10 μl volume was about 1500 pfu, at a

multiplicity of infection of 50, relative to the bacteria inoculated 1 h before. 50 larvae were used in each experiment. Larvae were placed into Petri dishes, incubated at 37°C in the dark, and lethality was monitored at 20 hpi. The mean and SD are based on two separate experiments. Significance was assessed by a χ^2 test performed on the pooled data ($\chi^2_{[1]} = 4.072$, $p = 0.0436$).

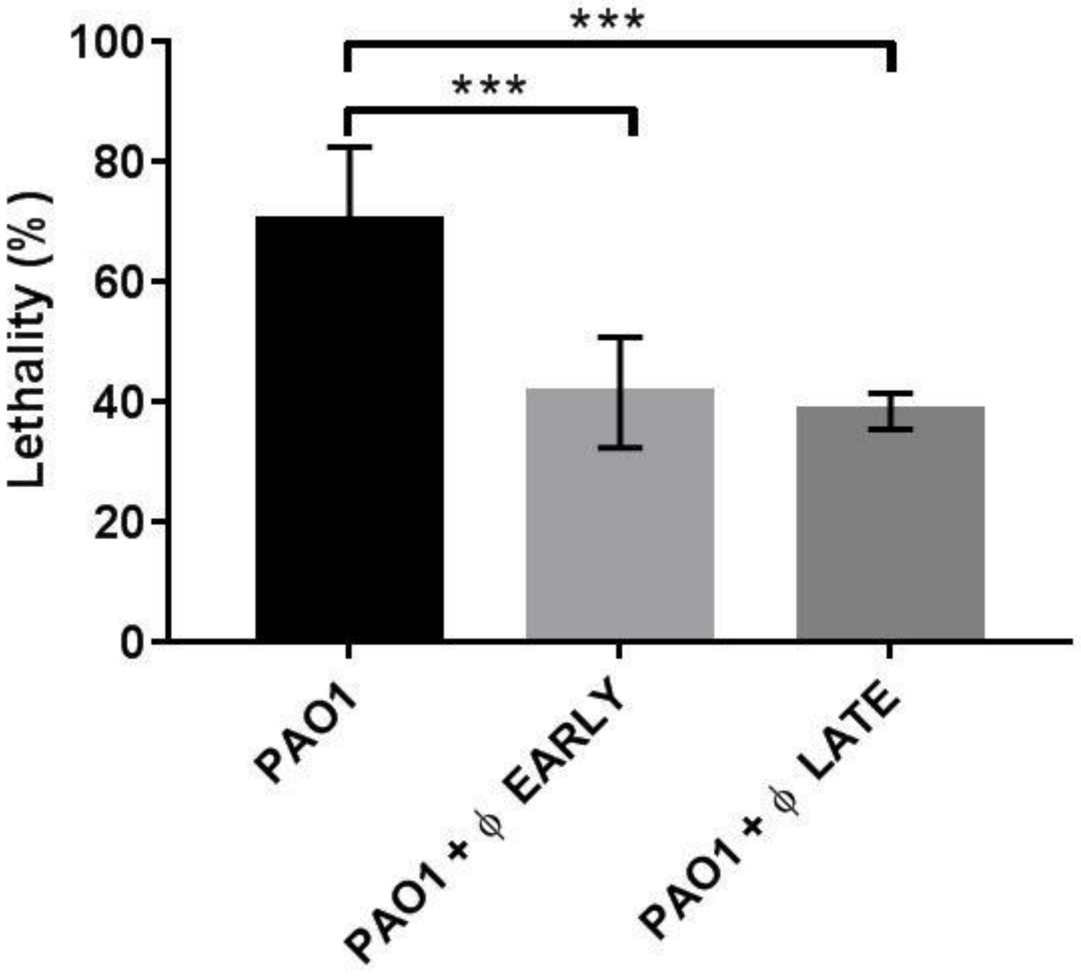

Fig. S3. Similar efficiency of early and late phage infection times against PAO1 infection in zebrafish embryos. An inoculum of 2 nl of PAO1, containing approximately 30-40 cfu/embryo, was injected in the yolk sac of 48 hpf embryos (PAO1). The phage cocktail (300-500 pfu/embryo) was then injected (PAO1+ϕ) at two different time points: about 30 min (EARLY) and 7 hours (LATE) after bacterial injection. A highly significant reduction in terms of embryo lethality was observed with either injection time: lethality was reduced from 70±12% to 42±9% and to 38±3% in early and late injection, respectively; no significant difference was present between the two time treatments. Significance was assessed by a χ^2 test performed on the pooled data (PAO1 vs PAO1+ϕ EARLY $\chi^2_{[1]} = 28.299$, $p = 1.04E10\text{-}7$; PAO1 vs PAO1+ϕ LATE $\chi^2_{[1]} = 15.546$, $p = 8.05E10\text{-}5$; PAO1+ϕ EARLY vs PAO1+ϕ LATE $\chi^2_{[1]} = 0.211$ $p = 0.646$.

Table S1.

PHAGE NAME	Gene Bank Accession Number	Taxonomy	Genome lenght (nt)	Presence of potentially harmful genes for therapy	Growth on PAO1	Latent period on PAO1	Burst size on PAO1 (pfu)
vB_PaeP_PYO2	MF490236	Caudovirales; Podoviridae; Lit1virus	72697	NO	+	20 min	200
vB_PaeP_DEV	MF490238	Caudovirales; Podoviridae; Lit1virus	72697	NO	+	20 min	120
vB_PaeM_E215	MF490241	Caudovirales; Myoviridae; P1virus; unclassified Punalikevirus	66789	NO	+	40 min	230
vB_PaeM_E217	MF490240	Caudovirales; Myoviridae; P1virus; unclassified Punalikevirus	66291	NO	+	30 min	250

Name and accession number of the four phages that compose the phage cocktail against *P. aeruginosa* PAO1 infections. The genome sequences of the phages are deposited in GenBank. Growth parameters have been described in Forti et al., 2018.

Video S1. PAO1-GFP microcolonies formation in the hindbrain ventricle of CF zebrafish embryos. Time laps imaging of bacterial proliferation following the injection in the hindbrain ventricle of 24 hpf CF zebrafish embryos.

Video S2. Efficacy of phage therapy in WT zebrafish embryos. In WT+PAO1 injected embryo (upper part), the fluorescence increased at 14 hpi, indicating that the GFP-positive bacteria multiply, whereas in the WT+PAO1+ϕ embryos (bottom part) the fluorescence after an initial increase at 10 hpi was reduced at later times (at 12-14 hpi), likely as an effect of the phage anti-bacterial activity.

Video S3. Efficacy of phage therapy in CF zebrafish embryos. In CF+PAO1 injected embryo (upper part), the fluorescence increased at 14 hpi, indicating that the GFP-positive bacteria multiply, whereas in the CF+PAO1+ϕ embryos (bottom part) the fluorescence after an initial increase at 10 hpi was reduced at later times (at 12-14 hpi), likely as an effect of the phage anti-bacterial activity. The amount of bacterial infection was higher in a CF background in comparison to WT (compare Video S2 with Video S3).
